# A novel strain of *Cellulosimicrobium funkei* can biologically detoxify aflatoxin B_1_ in ducklings

**DOI:** 10.1111/1751-7915.12244

**Published:** 2015-01-23

**Authors:** Lv-Hui Sun, Ni-Ya Zhang, Ran-Ran Sun, Xin Gao, Changqin Gu, Christopher Steven Krumm, De-Sheng Qi

**Affiliations:** 1 Department of Animal Nutrition and Feed Science, College of Animal Science and Technology, Huazhong Agricultural UniversityWuhan, Hubei, 430070, China; 2 Department of Animal Science, Cornell UniversityIthaca, NY, 14853, USA

## Abstract

Two experiments were conducted to screen microorganisms with aflatoxin B_1_ (AFB_1_) removal potential from soils and to evaluate their ability in reducing the toxic effects of AFB_1_ in ducklings. In experiment 1, we screened 11 isolates that showed the AFB_1_ biodegradation ability, and the one exhibited the highest AFB_1_ removal ability (97%) was characterized and identified as *Cellulosimicrobium funkei* (*C. funkei*). In experiment 2, 80 day-old Cherry Valley ducklings were divided into four groups with four replicates of five birds each and were used in a 2 by 2 factorial trial design, in which the main factors included administration of AFB_1_ versus solvent and *C. funkei* versus solvent for 2 weeks. The AFB_1_ treatment significantly decreased the body weight gain, feed intake and impaired feed conversion ratio. AFB_1_ also decreased serum albumin and total protein concentration, while it increased activities of alanine aminotransferase and aspartate aminotransferase and liver damage in the ducklings. Supplementation of *C. funkei* alleviated the adverse effects of AFB_1_ on growth performance, and provided protective effects on the serum biochemical indicators, and decreased hepatic injury in the ducklings. Conclusively, our results suggest that the novel isolated *C. funkei* strain could be used to mitigate the negative effects of aflatoxicosis in ducklings.

## Introduction

Aflatoxins (AF) are secondary fungal metabolites that are largely produced by the fungi *Aspergillus flavus* and *Aspergillus parasiticus* (Diaz *et al*., 2002). Among the various dangerous AF and their metabolites, aflatoxin B_1_ (AFB_1_) is the most toxic mycotoxin, having harmful hepatotoxic, mutagenic, carcinogenic and teratogenic effects on many species of livestock. It is also classified as a group one carcinogen [International Agency for Research on Cancer (IARC), 1987 ]. Unfortunately, AFB_1_ can easily contaminate various types of crops and is a very prevalent contaminant of maize-based food and feed all over the world (Wu and Guclu, 2012; Hamid *et al*., 2013). The feed contaminated by AFB_1_ can pose serious problems to the health and productivity of livestock and can therefore cause significantly economic losses (Rawal *et al*., 2010; Wu and Guclu, 2012).

Several physical and chemical detoxification methods used to control AFB_1_ have been to some extent successful, while most of them have major disadvantages including nutrients loss and high costs, which limited their practical applications (Varga *et al*., 2010; Jard *et al*., 2011). Thus, scientists have come to favor the biological method, which is utilization of microorganisms and/or their enzymatic products to remove AF through microbial binding and/or degradation of mycotoxins into less toxic compounds, giving a characterization of specific, efficient and environmentally sound detoxification (Wu *et al*., 2009; Guan *et al*., 2011).

Many studies have shown that AFB_1_ can be biologically detoxified by various species of microorganisms, including fungi, such as *Pleurotus ostreatus* (Motomura *et al*., 2003), *Trametes versicolor* (Zjalic *et al*., 2006), yeast such as *Trichosporon mycotoxinivorans* (Molnar *et al*., 2004) and *Saccharomyces cerevisiae* (Pizzolitto *et al*., 2013), and bacteria, such as lactic acid bacteria (Bagherzadeh Kasmani *et al*., 2012; Nikbakht Nasrabadi *et al*., 2013), *Stenotrophomonas maltophilia* (Guan *et al*., 2008), *Myxococcus fulvus* (Guan *et al*., 2010) and *Rhodococcus species* (Cserháti *et al*., 2013). Unfortunately, few of these microorganisms, their metabolites and/or degradation products have been utilized in animal feed due to a lack of information on the mechanisms of detoxification, the efficiency and stability of detoxification under different oxygen, pH or bile conditions, as well as their potential side-effects needs to be further investigated.

The objective of this study was to screen novel AFB_1_ biodegradation microorganisms that could be applied in feed industry. Because AFB_1_ is a furanocoumarin derivative which has a similar chemical structure to polycyclic aromatic hydrocarbons (PAHs) (Yu *et al*., 2004; Guan *et al*., 2008), we therefore hypothesized that the microorganism having biodegradable activity on PAHs maybe also have the same effect towards AFB_1_. We therefore chose soil samples around petroleum factories, which were contaminated with PAHs for the screening of the microorganisms that could biodegrade AFB_1_ (Pampanin and Sydnes, 2013). Since coumarin is the basic chemical structure of AFB_1_, along with the relatively safe and inexpensive characterization, we used medium-containing coumarin as the only carbon source to screen AFB_1_-biodegrading microorganisms by replicating the well-established method previously conducted (Guan *et al*., 2008). Since duckling is extremely sensitive to the toxic effects of AFB_1_ (Shi *et al*., 2013), and thus it was used to evaluate the detoxification effects of the isolate in this study. In this study, we successfully obtained the isolate *Cellulosimicrobium funkei* T_3_-5 (*C. funkei*), which exhibited excellent AFB_1_ biodegradation ability both *in vitro* and *in vivo*. Our findings suggested a feasible approach for a safe and efficient method to control AFB_1_ levels in the animal feed industry.

## Results

### Experiment 1

#### Screening for AFB_1_ biodegradation microbes

A total of 11 strains were isolated from three soil samples by coumarin medium, and all of them showed various degrees of ability to reduce concentrations of AFB_1_ in the liquid medium after 72 h incubation at 37°C (Table 1), which were calculated from the high-performance liquid chromatography (HPLC) results. The chromatograms of HPLC analysed results were shown in Fig. S2. Among the 11 screened strains, seven showed the potential of reducing AFB_1_ in the medium over 70%, and the isolate T_3_-5 was the most effective strain with an observed 94.2% AFB_1_ reduction in the medium (Table 1).

**Table 1:** Ability of AFB_1_ biodegradation by screened isolates^a^

Isolate^b^	AFB_1_ biodegradation (%)
T_1_-1	84.9 ± 4.0
T_1_-2	41.7 ± 4.1
T_1_-3	86.3 ± 7.3
T_1_-4	24.9 ± 3.8
T_2_-1	75.9 ± 1.8
T_3_-1	51.5 ± 6.9
T_3_-2	81.2 ± 4.9
T_3_-3	87.1 ± 4.0
T_3_-4	20.4 ± 4.3
T_3_-5	94.2 ± 2.4
T_3_-6	70.1 ± 4.5

a Values are expressed as means ± SD (*n* = 5).

b Isolates are screened from soil samples using coumarin as the only carbon source.

#### Identification of isolate T_3_-5

**Fig 1:**
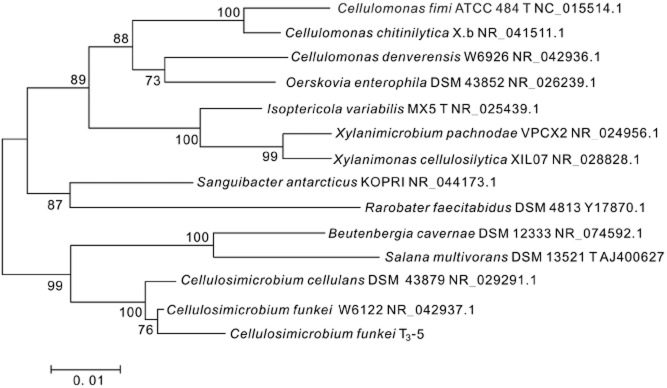
Neighbour-joining phylogenetic tree based on 16S rDNA gene sequences showing the relationships among the species of the genus *Cellulomonas* and related specifies. Bootstrap values calculated for 1000 replications are indicated. Bar, 2 substitutions per 100 nucleotides. Accession numbers from Genbank are given in brackets.

Microscopic morphological results showed that isolate T_3_-5 is a gram-positive bacterium, which appeared as circular, yellow, a smooth surface and an entire edge after 18 h of incubation at 37°C on the Luria Bertani (LB) agar (Fig. S1). Physiological and biochemical studies showed that the T_3_-5 strain was able to utilize most oligosaccharides including glucose, maltose, D-xylose, galactose, D-sorbitol, D-raffinose and as well as sucrose as a sole carbon source. The T_3_-5 strain could also hydrolyse cellulose, gelatin and Tween 80, but not amylum (Table 2). The 16S rDNA sequencing result of the isolate T_3_-5 [National Center for Biotechnology Information (NCBI) GenBank: KM032184] showed 99% deoxyribonucleic acid (DNA) sequence homology to that of *Cellulosimicrobium funkei* listed by NCBI (GenBank: NR_042937.1, Fig. 1). Taken together, the morphological, physiological, biochemical data as well as the NCBI blast results suggest that the isolate T_3_-5 belonged to *C. funkei*, which is an aerobic and facultatively anaerobic gram-positive bacterium. The above strain is deposited at China Center for Type Culture Collection (CCTCC) in Wuhan University, and has a preservation number of CCTCC NO: M 2013564.

**Table 2:** Biochemical and physiological characteristics of *C. funkei* T_3_-5

Characteristic	Result	Characteristic	Result	Characteristic	Result
Acid fermentation of:		Phosphatidylcholine	−	10% NaCl	−
Glucose	+	Tween 80	−	Acid medium	−
Maltose	+	Amylum	−	Other test:	
D-Xylose	+	Cellulose	+	Motility	−
Galactose	+	Xylan	−	Catalase test	+
D-Sorbitol	−	Nitrate reduction	+	Methyl red test	+
D-Raffinose	+	Organic acid	+	Urease test	+
Sucrose	+	Yeast cell	−	V-P test	−
Glycerol	+	Growth on:		Oxidase test	−
Hydrolysis of:		2%, 5% NaCl	+	Congo red tolerance	+
Gelatin liquefaction	+	7% NaCl	(+)		

+, Positive; −, negative; (+), weakly positive.

#### Characterization of AFB_1_ biodegradation by *C. funkei* T_3_-5

**Fig 2:**
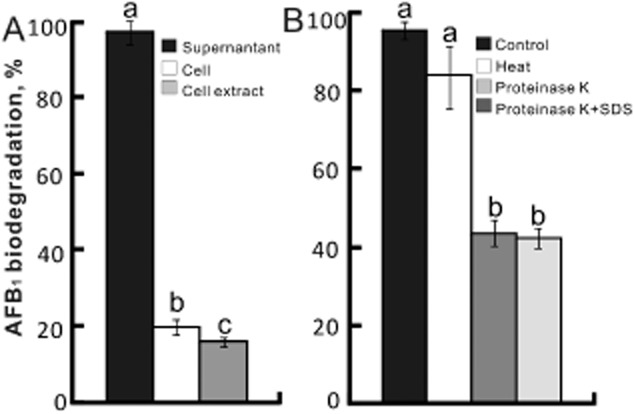
Ability of AFB_1_ biodegradation by culture supernatant, cell and cell extract of *C. funkei* after 72 h fermentation (A); and culture supernatant of *C. funkei* was determined by pretreating the supernatant by proteinase K with or without SDS or heat respectively (B). Values are means ± SD, *n* = 5. Bars without a common letter differ, *P* < 0.05.

The culture supernatant of *C. funkei* T_3_-5 showed the strongest (*P* < 0.05) AFB_1_ biodegradation ability compared with viable cell and cell extract, which removed 97%, 20% and 16% AFB_1_ after 72 h incubation respectively (Fig. 2A). These results indicated that the activity of AFB_1_ biodegradation occurred primarily within the culture supernatant of *C. funkei*. The AFB_1_ biodegradation ability of the culture supernatant of *C. funkei* was decreased (*P* < 0.05) 54% and 56% after treated with proteinase K with or without SDS, respectively, while it was only slightly decreased by heat treatment (Fig. 2B).

### Experiment 2

#### Performance

Non-significant differences in initial body weight were observed among the four groups (Table 3). After 2 weeks of experimental treatments, the growth performance was significantly affected by oral administration of AFB_1_ and *C. funkei* or their interactions (Table 3). Compared with the control, the final body weight (BW), overall daily BW gain and overall daily feed intake of ducklings were decreased (*P* < 0.05) 42%, 49% and 38%, along with increased (*P* < 0.05) 23% overall feed/gain ratio by AFB_1_ administration respectively. Although the AFB_1_ + *C. funkei* group showed the similar trend to AFB_1_ group that decreased (*P* < 0.05) the final BW (33%), overall daily BW gain (39%) and overall daily feed intake (39%) of ducklings respectively, administration of *C. funkei* prevented (*P* < 0.05) the loss in final feed conversion and BW (9%) at the first week. In addition, administration of *C. funkei* alone reduced (*P* < 0.05) the overall feed/gain ratio (9%), and did not affect the other growth performance parameters, when compared with the control. No mortality due to AFB_1_ administration was found in this study.

**Table 3:** Effects of administration of AFB_1_ and *C. funkei* on growth performance in ducklings^a^

	Control	AFB_1_^a^	*C. funkei*^c^	AFB_1_ + *C. funkei*
week 0 BW, g	119.3 ± 1.3	118.8 ± 1.0	118.5 ± 0.6	118.8 ± 0.5
week 1 BW, g	372.5 ± 30.0^a^	309.4 ± 7.0^c^	364.7 ± 16.2^a^	336.6 ± 16.4^b^
week 2 BW, g	786.9 ± 61.2^a^	458.5 ± 61.7^b^	858.8 ± 42.6^a^	527.8 ± 63.3^b^
week 2 BW gain, g/day	47.7 ± 4.3^a^	24.3 ± 4.4^b^	52.9 ± 3.0^a^	29.2 ± 4.5^b^
week 2 feed intake, g/day	89.7 ± 9.0^a^	55.5 ± 5.7^b^	90.3 ± 3.1^a^	55.1 ± 7.8^b^
week 2 feed/gain, g/g	1.88 ± 0.04^b^	2.32 ± 0.25^a^	1.71 ± 0.03^c^	1.89 ± 0.16^b^

a Values are expressed as means ± SD (*n* = 5), and means with different superscript letters differ (*P* < 0.05).

b Each duckling oral administrated 100 μg AFB_1_/kg BW per day.

c Each duckling oral administrated *C. funkei* at 10^8^ cfu/per day.

#### Serum biochemistry and liver histology

**Fig 3:**
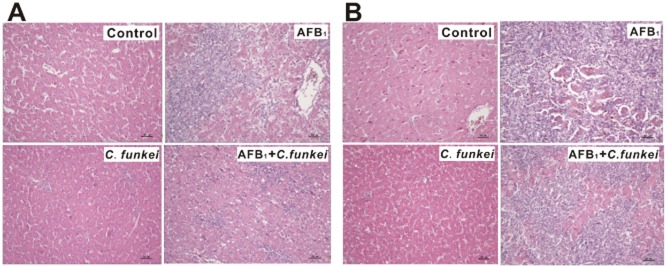
Photomicrographs of hepatic sections stained with haematoxylin and eosin (40× magnification) of ducklings from different treatment groups on (A) week 1 and (B) week 2 respectively.

The results showed that the serum biochemical and histological parameters were significantly affected by administration of AFB_1_ and *C. funkei* or their interactions (Table 4). The AFB_1_ administration led to increased (*P* < 0.05) activity of aspartate aminotransferase (AST; 404% and 867%) and alanine aminotransferase (ALT; 82% and 282%), along with decreased (*P* < 0.05) concentration of total protein (TP; 34% and –) and albumin (ALB; 44% and –) in the serum of ducklings at the first and second week respectively. Strikingly, AFB_1_ + *C. funkei* group decreased (*P* < 0.05) the activity of AST (43 and 44%) and ALT (28 and 20%), along with increased (*P* < 0.05) concentration of TP (22% and –) and ALB (42% and –) in serum of ducklings at the first and second week, respectively, compared with the AFB_1_ group (Table 4). Furthermore, the histological analysis results showed AFB_1_ administration-induced hepatic injury, such as vacuolar degeneration, necrosis and bile duct hyperplasia at the first week and increased liver damage on the second week. Notably, AFB_1_ + *C. funkei* group alleviated the liver damage was observed in the AFB_1_ group (Fig. 3).

**Table 4:** Effects of administration of AFB_1_ and *C. funkei* on serum biochemical parameters in ducklings^a^

	Control	AFB_1_^b^	*C. funkei*^c^	AFB_1_ + *C. funkei*
week 1				
ALT, U/l	46.5 ± 5.8^c^	234.8 ± 48.7^a^	48.3 ± 7.8^c^	132.8 ± 26.7^b^
AST, U/l	68.3 ± 8.4^c^	124.5 ± 5.4^a^	68.8 ± 11.3^c^	89.8 ± 8.1^b^
TP, g/l	29.4 ± 1.2^a^	19.4 ± 1.8^c^	28.9 ± 0.8^a^	23.6 ± 1.0^b^
ALB, g/l	13.5 ± 0.4^a^	7.6 ± 0.8^c^	13.4 ± 0.7^a^	10.8 ± 0.1^b^
week 2				
ALT, U/l	35.3 ± 10.2^c^	341.0 ± 52.0^a^	39.00 ± 11.0^c^	190.0 ± 43.3^b^
AST, U/l	53.5 ± 8.7^c^	204.5 ± 44.7^a^	54.3 ± 8.3^c^	162.8 ± 30.9^b^
TP, g/l	29.9 ± 1.7	–	30.0 ± 0.3	–
ALB, g/l	14.0 ± 0.9	–	13.9 ± 0.2	–

a Values are expressed as means ± SD (*n* = 5), and means with different superscript letters differ (*P* < 0.05).

b Each duckling oral administrated 100 μg AFB_1_/kg BW per day.

c Each duckling oral administrated *C. funkei* at 10^8^ cfu/per day.

–, Undetectable.

## Discussion

The two most novel findings from the present study were: (i) we successfully screened a novel AFB_1_ biodegradation microorganism *C. funkei* T_3_-5, and (ii) oral administration of *C. funkei* effectively alleviated the adverse effects induced by AFB_1_ in the ducklings. The *C. funkei* is a gram-positive, aerobic and facultatively anaerobic, non-spore-forming rod or coccus-shaped bacterium of the genus *Cellulosimicrobium*, consistent with previously reported (Brown *et al*., 2006). *In vivo*, *C. funkei* demonstrated effective AFB_1_ biodegradation ability that 97% of AFB_1_ can be removed after 72 h incubation. Interestingly, these reported values were much higher than those of previously reported various microorganisms, such as *Rhodococcus erythropolis* (67%, Alberts *et al*., 2006), *Flavobacterium aurantiacum* (74.5%, Smiley and Draughon, 2000), *Mycobacterium strain* (80%, Hormisch *et al*., 2004), *M. fulvus* (81%, Guan *et al*., 2010) and *S. maltophilia* (83%, Guan *et al*., 2008). Strikingly, Cserháti and colleagues (2013) found that several *Rhodococcus* species displayed more than 97% AFB_1_-degrading ability, along with effective degrading ability to other common mycotoxins also, which also offered a promising strategy to control mycotoxins. Moreover, our results implied that the compounds biodegrading AFB_1_ were mainly within the fermentation supernatant of *C. funkei* rather than in its viable cell and cell extract. Notably, the AFB_1_ biodegradation activity of fermentation supernatant of *C. funkei* was decreased more than 50% after treated with proteinase K or plus SDS, which is similar to the AFB_1_ biodegradation by the culture supernatant of *F. aurantiacum* and *S. maltophilia* reported earlier, and indicated the active ingredient could therefore be protein or perhaps an enzyme (Alberts *et al*., 2006; Guan *et al*., 2008). Furthermore, the AFB_1_ biodegradation activity was positively correlated with the protein content from the fermentation supernatant of *C. funkei* by ammonium sulfate precipitation (Table S1) which provides further evidence that the active ingredient could be protein (Callejón *et al*., 2014; Duong-Ly and Gabelli, 2014). However, heating may cause denaturation of proteins, strikingly, the AFB_1_ biodegradation activity of fermentation supernatant of *C. funkei* was only slightly affected by heating. It may be interpreted by (i) the protein involved in the AFB_1_ biodegradation is heat resistant (Wang *et al*., 2007), and (ii) the fermentation supernatant may contain cell wall, and heating may increase permeability of their external layer and lead to the increasing availability of the otherwise hidden binding sites for AFB_1_ (Shetty *et al*., 2007). Taken together, our results revealed that the active ingredient of AFB_1_ biodegradation in the fermentation supernatant of *C. funkei* could be enzyme and other active ingredients such as, cell wall. However, systematic identification of the active ingredients in the fermentation supernatant of *C. funkei* and its detoxification mechanism is still needed to be explored in the future.

Administration of AFB_1_ reduced the growth rate and efficiency of feed utilization of ducklings, which were in accordance with those in previous studies (Pasha *et al*., 2007). The negative effects of AFB_1_ on feed intake, BW gain and the feed conversion have been associated with anorexia, reluctance and inhibition of protein synthesis and lipogenesis (Bagherzadeh Kasmani *et al*., 2012). The present study showed that supplementation of *C. funkei* at dose of 10^8^ cfu/day prevented the loss in feed conversion throughout. Meanwhile, although supplementation of *C. funkei* had no significant effect on the final BW, while improved body weight was observed during the first week. The beneficial effects of supplementation of *C. funkei* could be due to (i) toxin biodegraded by *C. funkei in vivo* was evidenced by a 97% removal of AFB_1_, and (ii) the *C. funkei* could hydrolyse cellulose and improved the cellulose utilization. In addition, no adverse effects in productivity parameters were found between ducklings in control group and the experimental group administered *C. funkei* alone, indicating that *C. funkei* was non-toxic and safe. Similar results were obtained from recent studies that *Nocardia corynebacteroides* and *S. cerevisiae* can partly detoxify chicken feed contaminated with AFB_1_ (Tejada-Castañeda *et al*., 2008; Pizzolitto *et al*., 2013).

Activities of serum enzymes such as ALT and AST, and concentrations of serum TP and ALB have been described as valuable parameters of hepatic injury and function (Bagherzadeh Kasmani *et al*., 2012; Lv *et al*., 2014). Administration of AFB_1_ alone increased ALT and AST activity, along with decreased TP and ALB concentrations compared with the control diet. These outcomes were consistent with previous studies, which provided evidence that liver injury was induced by AFB_1_ (Bagherzadeh Kasmani *et al*., 2012; Shi *et al*., 2013). Results obtained from the present study showed that serum biochemical changes could be ameliorated by *C. funkei* administration. Moreover, histopathological changes in the livers of ducklings exposed to AFB_1_ were similar to those reported on avian aflatoxicosis (Denli *et al*., 2009). Administration of *C. funkei* showed stronger protective effect on the histopathological changes on the first week, but consistent with the growth performance results, was unable to prevent liver injury on the second week. This may be due to (i) the duckling-ingested AFB_1_ was added along with the increase in BW, while the *C. funkei* (10^8^ cfu/day) dosage was not changed, and (ii) the toxic potency of AFB_1_ was increased due to the prolonged exposure times (Centoducati *et al*., 2009) and finally beyond the detoxification capacity of *C. funkei*. Since ducklings exposure to AFB_1_ from the naturally contaminated feed is usually much lower (at least 10 times) than the administration of AFB_1_ at 100 μg/kg BW per day in our study (Yang *et al*., 2012), supplementation of *C. funkei* may therefore exert better protective effects on aflatoxicosis in practice.

Although *C. funkei* has showed potent AFB_1_ biodegradation capability and safety *in vivo* study, directly using this microbe as a feed additive seems challenged by the fact that *C. funkei* is an opportunistic pathogen (Petkar *et al*., 2011). Therefore, our ongoing research was focus in two directions: (i) exploring the mechanism of AFB_1_ biodegradation by *C. funkei*, which try to separate the enzyme and/or other active ingredients such as cell wall that could biodegradation the AFB_1_ and (ii) using *C. funkei* alone or with other microbial to do the solid-state fermentation on rapeseed meal and cottonseed meal to improve crude fiber digestibility, reduce AFB_1_ contents and produce AFB_1_ biodegradation active ingredients in these feedstuffs.

In summary, the *C. funkei* isolated in the present study, exhibited significant improvements in the capabilities of biodegradation of AFB_1_ *in vitro*. Moreover, an *in vivo* study verified its AFB_1_ biodegradation activity in ducklings with regard to partial improvement growth performance, serum biochemistry, hepatotoxicity and histopathology of livers. Additionally, the *in vivo* study showed that administration of *C. funkei* at 10^8^ cfu/day was non-toxic and safe to administer to ducklings. Overall, these findings suggest that the use of *C. funkei* in AFB_1_-contaminated feed offers a new strategy to reduce the adverse effects of aflatoxicosis in ducklings.

## Experimental procedures

### Experiment 1

#### Soil samples and AFB_1_ biodegradation microorganism isolation

Three soil samples designated as T_1_, T_2_ and T_3,_ were collected around the factories of SINOPEC Wuhan Company, Shandong Jinqiao Coal Mine Company and Shandong Yankuang International Coking Company. All these samples were air-dried at room temperature. Microorganisms that could use coumarin as the only carbon source were then isolated from soil samples by using a standard procedure with minor modifications (Guan *et al*., 2008). Single colonies that were able to grow on the coumarin (Sigma Chemical Co., Bellefonte, USA) plate were selected for AFB_1_ biodegradation activity analysis according to the protocol described by Guan and colleagues (2008) with minor modifications. Initially, candidate isolates were cultured at 37°C in LB medium for 72 h, and then 950 μl fermented supernatant was taken and mixed with 50 μl 10 μg/ml AFB_1_ solution (Sigma Chemical Co., Bellefonte, USA) in a sterilized centrifuge tube, and then biodegradation tests were conducted at 37°C for 72 h. Finally, the reaction solution was centrifuged at 10 000 *g* at 4°C for 10 min to remove cells and the supernatant, and then it was collected for AFB_1_ quantification (Guan *et al*., 2008). The AFB_1_ concentration was determined by HPLC (Teniola *et al*., 2005) with a minor modification. AFB_1_ was extracted three times with chloroform from liquid cultures and cell-free extracts. The chloroform was evaporated under nitrogen gas, and the samples were dissolved in methanol, filtered by 0.22 μm filters for HPLC analysis. HPLC analysis was performed on a Shimadazu LC-20A binary gradient liquid chromatography equipped with a 5 μm × 4.6 mm × 250 mm C-18 reverse-phase column (ZORBAX Eclipse XDB-C18, Agilent). The mobile phase was acetonitrile/methanol/water (1:1:2, v/v/v) at a flow rate of 1 ml/min, and the sample temperature was set at 30°C. AFB_1_ was measured by UV (365 nm.) detector (Shimadazu SPD-20A). The sterilized LB medium alone substituted fermentation supernatant incubated with AFB_1_ solution was used as the negative control.

#### Identification of the AFB_1_ biodegradation isolate

Total DNA was extracted from the AFB_1_ biodegradation isolate using TIANamp Bacterial DNA Kit (Tiangen Biotech, Beijing, China) according to the manufacturer's instructions. The forward primer (27f: 5′-GAGAGTTTGATCCTGGCTCAG-3′) and the reverse primer (1492r: 5′-CTACGGCTACCTTGTTACGA-3′) were used to amplify the 16S ribosomal (r)DNA (Minerdi *et al*., 2012). After the amplified 16S rDNA fragment was purified using the Gel Extraction Kit, it was ligated into the pMD18-T vector, and transformed into the *Escherichia coli* JM109 strain by calcium chloride activation (Dagert and Ehrlich, 1979). The positive colonies were selected for DNA sequencing (Tsingke, Wuhan, China). The obtained DNA sequence and NCBI GenBank-derived sequences were aligned using the ClustalX program (Thompson *et al*., 1997). Neighbour-joining phylogenetic tree and bootstrap values were analysed by the mega program (Tamura *et al*., 2013). Physiological and biochemical tests were carried out following the method described by Holt and colleagues (1994).

#### Characterization of AFB_1_ biodegradation activity of *C. funkei* T_3_-5

After *C. funkei* T_3_-5 grew at 37°C in LB medium for 72 h, then the cell, cell extract and fermentation supernatant were prepared as previously described (Guan *et al*., 2008), and their AFB_1_ biodegradation ability was tested as described before. Specifically, supernatant was obtained by centrifuging fermentation supernatant at 12 000 *g* at 4°C for 20 min; cell was collected after being centrifuged at 12 000 *g* at 4°C for 20 min and washed twice with phosphate buffer (50 mM; pH 7.0); cell extract was produced by using ultrasonic cell disintegrator on ice, and the suspension was centrifuged at 12 000 *g* for 20 min at 4°C, and then it was filtered by 0.22 μm pore size sterile cellulose pyrogen free filters. Since the main active ingredients for AFB_1_, biodegradation were found within the fermentation supernatant, further assessment was conducted through *in vivo* experiments. The AFB_1_ biodegradation stability of fermentation supernatant of *C. funkei* was determined by the residual activity after the supernatant was treated by proteinase K (0.5 mg/ml) with or without SDS (5.0%) at 37°C for 6 h, or boiled at 100 °C for 15 min respectively. The untreated fermentation supernatant of *C. funkei* was used as the positive control.

### Experiment 2

#### Ducklings, treatments and samples collection

Our animal protocol was approved by the Institutional Animal Care and Use Committee of Huazhong Agricultural University, China. A total of 80 day-old Cherry Valley commercial ducklings were randomly divided into four treatment groups with four replicates of five birds each. The trial was arranged in a 2 by 2 factorial design that included oral administration of AFB_1_ or solvent and *C. funkei* or solvent respectively. All birds were allowed free access to a similar corn-soybean meal diet (Shi *et al*., 2013) and distilled water ad libitum. The LD_50_ of AFB_1_ in duckling is 2.8 mg/kg BW (Yunus *et al*., 2011), and we chose 100 μg/kg BW since the dose of subchronic toxicity test was chosen between 1/10-1/50 LD_50_ (Jin *et al*., 2008). After 3 days of acclimation, each group was administered an oral dose of AFB_1_ [dissolved in 1.0% dimethylsulphoxide (DMSO)] at 100 μg/kg BW or an equivalent amount of sterile DMSO, along with an administration of 1 ml 10^8^ cfu/ml *C. funkei* or an equivalent amount of sterile LB medium per day respectively. The administration continued for 2 week. Birds were monitored mortality daily, along with body weight and feed intake measured weekly. Meanwhile, four birds from each treatment group were slaughtered weekly to collect blood and liver for the preparation of serum, and liver histological tissue samples were prepared as previously described (Shi *et al*., 2013; Sun *et al*., 2013).

#### Serum biochemical and histological analysis

The serum activities of ALT and AST, along with concentrations of TP and ALB were determined in serum samples. Analyses of the serum samples were measured by an automatic biochemistry analyser (Beckman Synchron CX4 PRO, CA, USA). The liver tissues were fixed in 10% neutral buffered formalin and processed for paraffin embedding, sectioned at 5 μm and stained with haematoxylin and eosin, by standard procedure (Pizzolitto *et al*., 2013). Liver sections from all birds were microscopically examined.

#### Statistical analysis

Data generated from experiment 1 were analysed by one-way ANOVA to test the main effects of AFB_1_ biodegradation activity of *C. funkei*. Data generated from experiment 2 were analysed by two-way ANOVA to test the main effects of administration AFB_1_ and *C. funkei*. The Bonferroni *t*-test was followed for multiple mean comparisons if there was a main effect. All analyses were conducted using sas 8.2 (sas Institute). Data were presented as means ± SD, and significance level was set at *P* < 0.05.

## Supporting Information

**Fig. S1.** Morphology of (A) colony and (B) gram staining of *C. funkei* T_3_-5. **Fig. S2.** The selected chromatogram of HPLC, (A) 25 ug/kg AFB_1_ standard; (B) negative control; (C) positive control; (D) after AFB_1_ biodegradation by *C. funkei*. AFB_1_ biodegradation (%) = (C_peak area_ − D_peak area_)/ C_peak area_ × 100%. **Table S1.** Ability of AFB_1_ biodegradation by the protein from the culture supernatant of *C. funkei* by ammonium sulfate precipitation^1^.
