# Supplementary material for: A novel strain of Cellulosimicrobium funkei can biologically detoxify aflatoxin B1 in ducklings
Source: Microb Biotechnol. 2015 Jan 23;8(3):490–8. doi: 10.1111/1751-7915.12244 (PMC4408181; doi:10.1111/1751-7915.12244)
Supplement: Supplementary file 1 [file mbt20008-0490-sd1.doc]

**Online Supporting Material**

**Supplemental Table 1** Ability of AFB1 biodegradation by the protein from the culture supernatant of *C. funkei* by ammonium sulfate precipitation1

| **Ammonium sulfate** | **Protein, mg/ml^2^** | **AFB_1_ biodegradation, %** |
| --- | --- | --- |
| 40% | 0.012±0.01^c^ | 2.68±0.94^d^ |
| 50% | 0.028±0.01^c^ | 7.21±1.62^c^ |
| 60% | 0.158±0.03^b^ | 39.56±3.41^b^ |
| 70% | 0.204±0.02^a^ | 53.61±4.13^a^ |
| 80% | 0.157±0.01^b^ | 36.74±1.82^b^ |

^1^ The 50 mL culture supernatants of *C. funkei* were collected and precipitated by 40-80% ammonium sulfate, and then the precipitated protein dissolved in 4 mL PBS buffer and dialyzed by 3500 Da dialysis bag. Finally, 950 µL dialyzed samples was mixed with 50 µL 10 µg/mL AFB_1_ solution for the biodegradation tests as previously described. Values are expressed as means ± SD (*n* = 5), and means with different superscript letters differ (*P* < 0.05).

^2^ Protein are the dialyzed samples.

**Online Supporting Material**

**
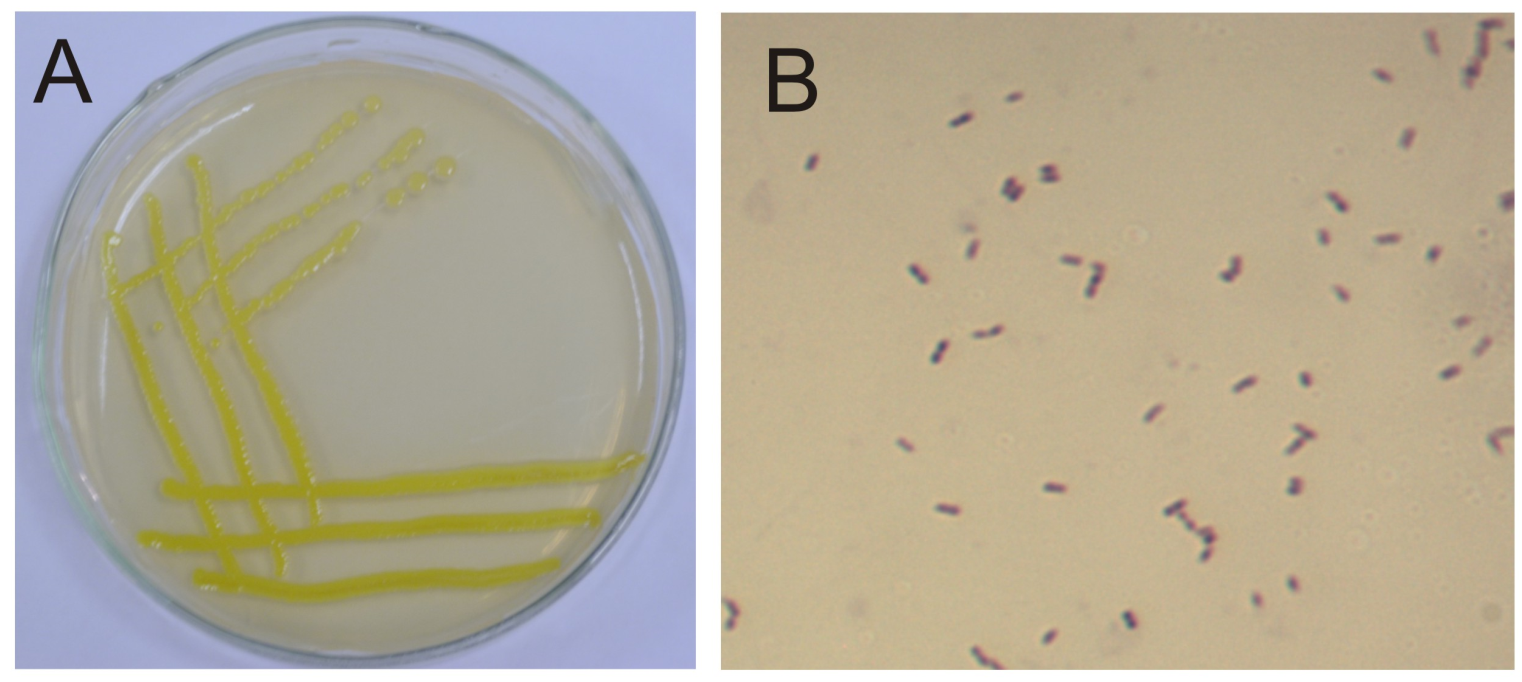
**

**Supplemental Fig. 1.** Morphology of (A) colony and (B) gram staining of *C. funkei* T_3_-5.

**Online Supporting Material**

**
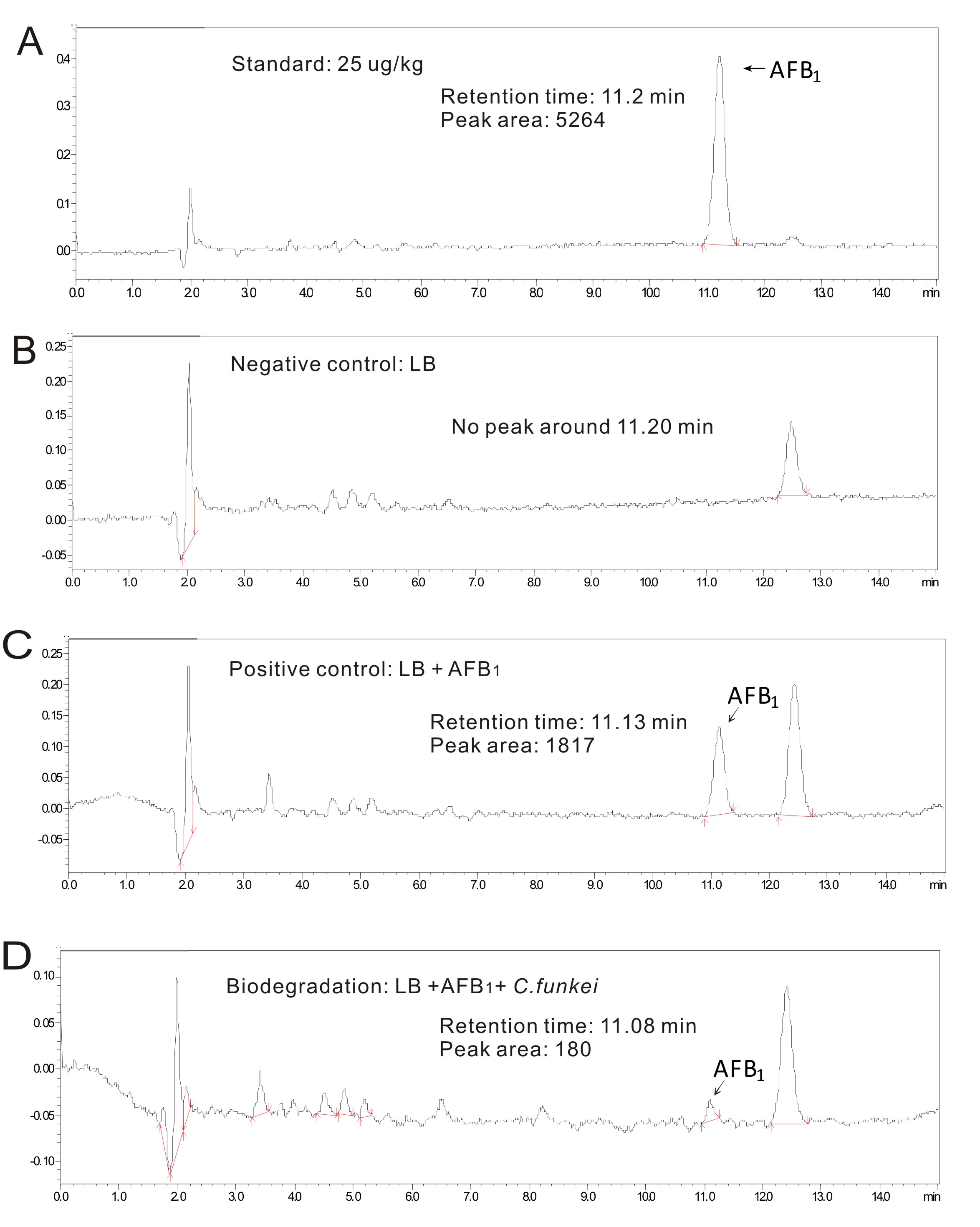
**

**Supplemental Fig. 2.** The selected chromatogram of HPLC, (A) 25 ug/kg AFB_1_ standard; (B) negative control; (C) positive control; (D) after AFB_1_ biodegradation by *C. funkei*. AFB_1_ biodegradation (%) = (C_peak area_ - D_peak area_)/ C_peak area_ × 100%.
